# Cognitive-pragmatic functions of mitigation in therapeutic conversations emphasizing rapport management

**DOI:** 10.3389/fpsyg.2023.1114146

**Published:** 2023-02-13

**Authors:** Lulu Cheng, Haoran Mao, Ting Zhang

**Affiliations:** ^1^School of Foreign Studies, China University of Petroleum (East China), Qingdao, China; ^2^Shanghai Center for Research in English Language Education, Shanghai International Studies University, Shanghai, China; ^3^School of Foreign Studies, Shanxi Technology and Business College, Taiyuan, Shanxi, China

**Keywords:** mitigation, therapist-client conversations, rapport management theory, cognitive-pragmatic function, communicative strategy

## Abstract

Based on conversations between 15 clients and 5 therapists in the context of daily treatment, this study investigated therapist-client linguistic mitigation in a natural setting. The study found that (1) the therapists and clients mainly used three major types of mitigation, among which illocutionary mitigation and propositional mitigation were employed more frequently. Furthermore, direct dissuasion and disclaimers, as subtypes of mitigators, were the most regularly employed by therapists and clients, respectively. (2) Through cognitive-pragmatic interpretation under rapport management theory, it was found that mitigation in the therapist-client conversations mainly performed cognitive-pragmatic functions in giving the means to preserve positive face, maintaining social rights and concentrating on interactive goals, which were interpenetrated with each other in therapeutic conversations. (3) This study proposed that three cognitive-pragmatic functions jointly devoted to a rapport in therapeutic relationship to reduce therapeutic risk of conflicts.

## Introduction

As one of the crucial communicative strategies, mitigation is a central issue in interpersonal interaction, and much attention has been drawn to its significant role in establishing harmonious interpersonal relationships (Fraser, 1980; Holmes, 1984; Brown and Levinson, 1987; Caffi, 1999, 2007; Sbisà, 2001; Leech, 2014). Relative studies illustrate that mitigation in quite a few aspects, such as gender differences, institutional discourse and educational discourse, could maintain and improve interpersonal relationships (Lakoff, 1975; Caffi, 1999; Martinovsky, 2006; Li, 2008; Flores-Ferrán, 2010; Peng, 2018; Yang, 2018). Among the above studies, mitigation in therapist-client conversations has had broadened appeal since harmonious interpersonal communications in medical fields became a heated topic in social language studies for several decades. Much work has been done to examine the functions and significance of mitigation in therapist-client conversations (Fraser, 1980; Caffi, 1999; Delbene, 2004; Huo, 2004; Flores-Ferrán, 2010, 2012; Zhao, 2019). These studies have revealed that mitigation exerts a positive influence in therapeutic interactions by weakening illocutionary force; that is, mitigation could indirectly strengthen the pragmatic effects of therapist-client conversations by relieving the unwelcoming effects of speech acts with the purpose of pacification, solidarity building and persuasion (Li and He, 2016).

It is believed that the utilization of mitigation is beneficial to the rapport between therapists and clients, which can ease therapist-client tension and enhance their interpersonal relationships. Much work has been done to examine the relationship between mitigation and interpersonal relationships in disparate institutional interaction (Martinovsky, 2006; Liu, 2012; Yang, 2018; Bates, 2021; Dada and Onyas, 2021; Ergül, 2021). Nevertheless, examining cognitive-pragmatic functions and their role in mitigation has been rarely done from the perspective of rapport management, especially in therapeutic settings. Using the method of discourse analysis, this study attempts to analyze what types of cognitive-pragmatic function therapist-client mitigation performs and how they are achieved in various therapeutic settings.

## Previous research on mitigation

With the purpose of better illustrating how therapists and clients use mitigation for relationship management, it is necessary to offer a general account of the definitions of mitigation in interpersonal interaction. Fraser (1980) defined mitigation as modification of a speech act that reduced the unwelcoming effects that the act might have on its hearer. Holmes (1984) argued that mitigation was considered a more general communicative strategy for modifying, attenuating and boosting the illocutionary force of speech acts. Caffi (1999, 2007) treated mitigation as a kind of meta-linguistic awareness, with the help of which speakers could make their utterance and performance more effective. Both Holmes and Caffi supplemented Fraser’s definition by adding psychological factors. Although those definitions highlighted disparate domains, they all believed that mitigation had an impact on the effectiveness of illocutionary force by alleviating the unwelcoming effects of speech acts. Based on previous studies, this study attempts to define therapist-client mitigation as a communicative and pragmatic strategy used by therapists and clients to soften illocutionary force and reduce the unwelcoming effects of speech acts to better manage their interpersonal relationships.

The current studies on mitigation were classified into two aspects. One was about theories, while the other was about practice. For theoretical studies, the definition, classification and functions of mitigation have attracted wide attention. Fraser (1980) examined mitigation for the first time and suggested that mitigation itself was not a speech act but involved a certain effect that arose from a given speech act. Afterward, many scholars put forward various definitions of mitigation from disparate cognitive domains (Fraser, 1980; Holmes, 1984; He, 1985; Caffi, 1999; Ran, 2004; Martinovksi et al., 2005; Li, 2008). Although their points and concerns are different, most of them claimed that mitigation, as a vital communicative and pragmatic strategy, performed a crucial function in mitigating communicative nervousness and embarrassment through activating empathy.

For the classification, increasing subtypes of mitigation were proposed in various contexts (Fraser, 1980; Ran, 2004; Li, 2008). Fraser (1980) classified therapist-client mitigation into self-serving mitigation and altruistic mitigation, while Ran (2004), attaching importance to the linguistic particle mitigator “*ba*” and its pragmatic function, proposed six types of mitigation, which were indirect speech act, vagueness-oriented discursive structure, uncertain statement, inserted verb, disjunctive question and linguistic hedge. Afterward, Li (2008) generalized and concluded three major types and 14 subtypes of mitigation in conversations in TV interviews. Although disparate subtypes of mitigation vary in contexts and technical terms, most of them took the perspective of its pragmatic and interpersonal functions. In this article, with reference to the classification of Li (2008), mitigation was sorted into three types: propositional mitigation, covering understaters, evidentials, tag questions, and subjectivizers; illocutionary mitigation, including politeness markers, terms of address, supportive tags, and disclaimers; and perlocutionary mitigation, incorporating simple anticipation, concern showing, penalty taking and direct dissuasion.

Furthermore, emotional functions and interpersonal functions of mitigation were discussed frequently as a function of various discourses (Caffi, 1999; Schneider, 2010; Li, 2012; Li and He, 2016). Emotional functions concentrated on the psychological effects of mitigation. In particular, Caffi (1999) proposed that psychological distance showed a great impact on mitigation from the perspective of psychology and cognition. Thereafter, Li (2012) illustrated that mitigation exhibited empathic properties, which were perspective-taking, emotional convergence and altruism. It was found that emotional functions showed that speakers and hearers took advantage of mitigators to express their positive emotions and weaken the unwelcoming effects of illocutionary force to enhance inclusivity. While interpersonal functions featured in interpersonal relationships by giving face and being polite based on face-saving theory and the politeness principle (Brown and Levinson, 1987; Sbisà, 2001; Leech, 2014). Interpersonal functions of mitigation embodied broader meanings in interpersonal communication by controlling the rapport distance between speakers and hearers by emphasizing psychological avoidance, pacification and solidarity building (Schneider, 2010; Li and He, 2016). To better understand of how both functions of mitigation discussed above have impacts on pragmatic cognition practice, in this study, we concentrate more on cognitive-pragmatic functions in therapist-client mitigation.

For practice studies, mitigations of gender differences, institutional discourses and classroom discourses have inspired manifold discussions in recent years (Caffi, 1999; Wouk, 1999; Martinovsky, 2006; Li, 2008; Flores-Ferrán, 2010; Liu, 2012; Yang, 2018; Bates, 2021; Dada and Onyas, 2021; Ergül, 2021). Particularly, in the wake of the social turn in language studies, mitigation in institutional discourses has received intensive attention as more studies gave priority to disparate registers in social issues. Mitigation in therapist-client conversations, as the nucleus in therapeutic practice, has attracted wide attention among skyrocketing numbers of medical conflicts (Pamela, 2013). Therapist-client mitigation was regarded as a vital communicative and pragmatic strategy to accomplish interactive goals and establish rapport in therapist-client relationships (Caffi, 1999; Delbene, 2004; Huo, 2004; Flores-Ferrán, 2010, 2012; Zhao, 2019; Liu, 2020). Delbene (2004) conducted a case study in the context of a socially stigmatized disease and proposed that mitigation was applied the most when communicating with clients who suffered from a communicable disease that endangered public health. Flores-Ferrán (2010) investigated psychotherapist-client politeness in their conversations and mainly discussed the interaction between mitigation and indirect speech acts. Afterward, he investigated mitigation phenomena (e.g., bushes, shields) and indirectness in Spanish in an institutional setting to determine whether these are pragmatically motivated (Flores-Ferrán, 2012). Zhao (2019) concentrated on mitigation by Chinese doctors in online medical consultation and found that perlocutionary mitigation applied the most. Obviously, the majority of the studies on therapist-client mitigation were conducted and concentrated either on the side of therapists or of clients. Nevertheless, they were less helpful in determining why and how it was done to both therapists and clients because they put emphasis on one side of mutual communication instead of interactions.

Regarding the interpretation of frameworks of applied mitigation in therapist-client conversations, various studies worked with detailed cases or data analysis to describe or generalize its specific pragmatic functions or interpersonal effects to figure out general rules of mitigation in therapeutic sessions (Delbene, 2004; Huo, 2004; Flores-Ferrán, 2010; Zhao, 2019). Those descriptive studies about therapist-client discourses were far more similar to exploratory studies that deeply explored the connotations and cognitive-pragmatic functions in therapist-client mitigation (Wu, 2021; Yao et al., 2022; Zeng et al., 2022). Much remained relatively under investigated in the cognitive-pragmatic functions of mitigation in therapeutic conversations when it was illustrated from top to bottom, which meant theoretic foundation was employed to interpret inner rules of therapist-client conversations by interpersonal relationship theory with deductive reasoning rather than conclusions from generalization. Therefore, this study interpreted cognitive-pragmatic functions of therapist-client mitigation in interpersonal management based on the theoretical framework of Rapport Management Theory (RMT). Proposed by Spencer-Oatey, RMT incorporates the management of face, which is inclusive of the quality face and the social identity face, the management of rights and obligations and the management of interactional goals. It contains five important and interrelated domains covering the illocutionary domain, discourse domain, participation domain, stylistic domain and nonverbal domain. Three factors that influence management goals above are rapport orientation, contextual variables, and pragmatic principles and conventions (Spencer-Oatey, 2002, 2004, 2005, 2008).

In conclusion, there are relatively plentiful theoretical and descriptive studies about mitigation, and due to the lack of authentic materials, there are few in-depth empirical studies and applied studies, especially for therapist-client mitigation in real therapeutic sessions. Furthermore, most previous studies on therapist-client mitigation have explored either theoretical research or one-sided communications, while fewer exploratory studies have investigated on-the-spot, interdiscursive therapist-client conversations under the framework of interpersonal relationship management from top to bottom. Proceeding from the existing study, the current research aims to enhance the investigation of therapist-client mitigation and provide support for the construction of a rapport management-based therapist-client relationship by addressing its cognitive-pragmatic functions.

## Data and methods

The therapist-client conversations in this study were collected from two top hospitals in Beijing and mainly accumulated from the Thyroid Department, Hepatobiliary Department and Intestinal Department from June to August 2019, which included natural settings covering outpatient departments, inpatient wards and doctor duty rooms. The data collection was facilitated by therapists using digital voice recorders with the approval of both therapists and clients and those collected contents were mainly related to inquiries and diagnoses. The length of the audio recordings is 33 h in total. Furthermore, unrelated third-party conversations were removed, and only therapist-client conversations were retained. Eventually, the data in this study were comprised of the verbatim transcription of the audio recordings totaling up to 90,000 Chinese characters involving 5 therapists and 15 clients. Among the 5 therapists, 3 were male, and 2 were female. Among the 15 clients, 7 were male, and 8 were female. In all conversational data extracts, therapists are referred to as TH and clients as CL, and all mitigators are underlined and in bold. The transcript is processed in three steps. The first line of data is presented in Chinese Pinyin. In the second line, the utterance is a word-by-word gloss which will help the reader to know what is happening in the Chinese original. In the third line, there is the idiomatic translation of the original Chinese. Thus, the transcript has three lines which include the Chinese Pinyin, a word-by-word gloss, and an idiomatic translation. In consideration of the interlinguistic and intercultural discrepancies in transcript translation and the clinical setting, two therapists from Shanxi Provincial People’s Hospital and Shanxi Bethune Hospital and two professional translators in medical translation were invited to polish and finalize the transcript translation through panel discussions.

In this study, three steps were employed to determine the raw materials. First, Cool Edit Pro 2.1 was employed to remove unrelated third-party conversations from the entire 33-h audio recordings. Additionally, professional voice-to-text transcription software Luyinla (v8.0) was applied to transcribe dialogs which were repeatedly listened to and read to ensure the accuracy of the transcription. Finally, the definitions and classifications of mitigation were illustrated in great detail to therapists and translators. Afterward, a therapist and a translator worked as a group to interpret, analyze and translate Chinese into English. Two versions of translation were discussed, particularly for those uncertain or discrepant parts related to mitigation. The final version was settled by agreement among parties upon consultation. This study identified 24 instances, among which 58 migitators were marked in the targeted therapist-client conversations with 34 were said by therapists and 24 by clients.

## Cognitive-pragmatic functions of mitigation

**Figure 1:**
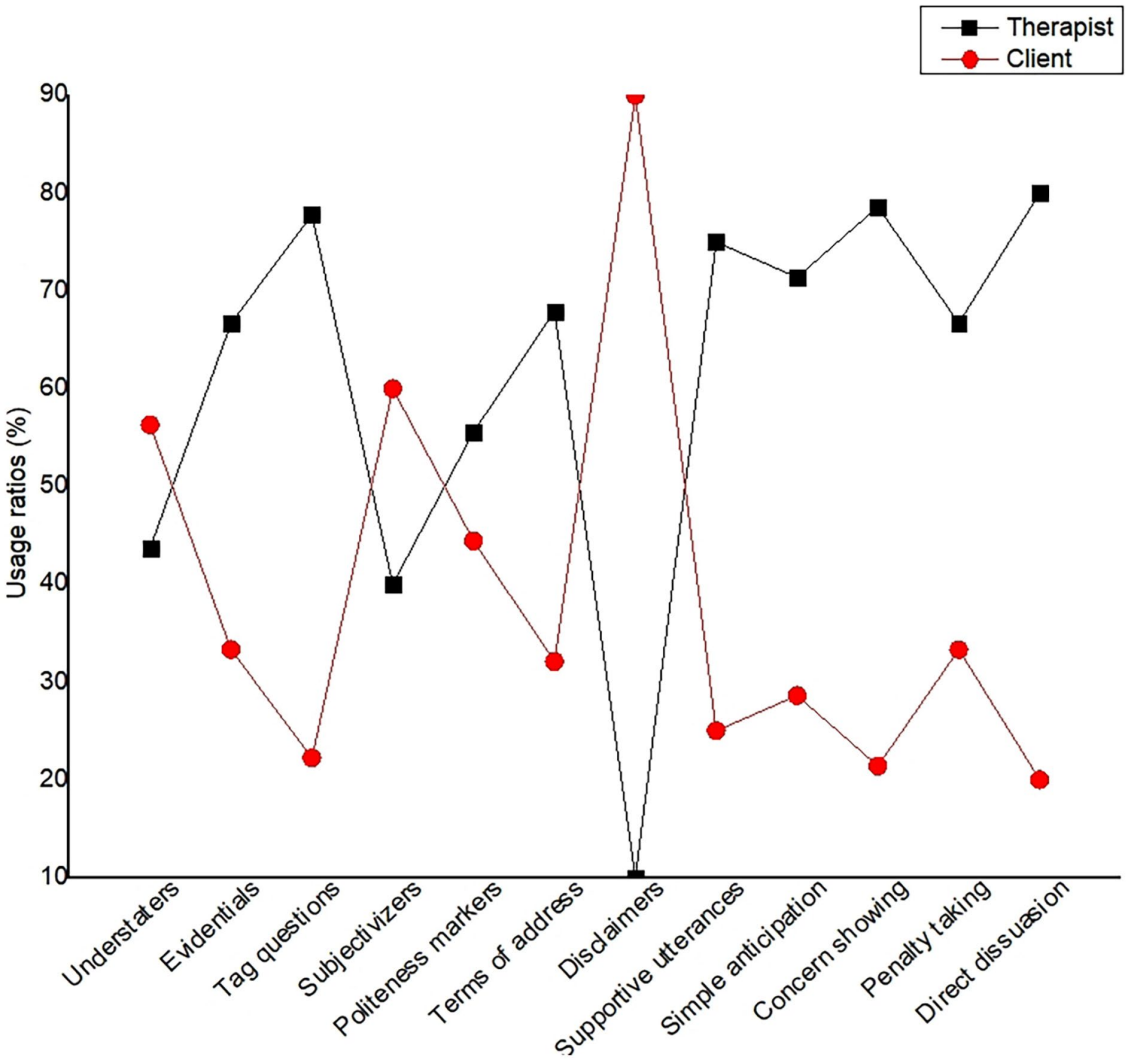
Usage ratios for subtype of mitigation for therapists and clients.

To maintain the rapport between therapists and clients, three types of mitigation were applied: propositional mitigation, illocutionary mitigation and perlocutionary mitigation, which all contain four subtypes. Table 1 shows examples and disparate usage ratios of the major three types and 12 subtypes of therapeutic mitigation. Figure 1 presents the disparate usage ratios of the 12 subtypes of mitigation by therapists and clients. Both Table 1 and Figure 1 represented the whole mitigation information in therapeutic sessions, which demonstrates its patterns and ratios in Chinese context. While conversational data extracts discussed in the following passages were the most illustrative representatives of cognitive-pragmatic functions for mitigation from the view of rapport management.

**Table 1:** Examples and ratios for therapist-client mitigation.

Type		Main expressions	Ratio
Propositional mitigation	Understaters	*稍微 (a bit)*, *有点儿 (a little)*, *有些 (some)*, *好像 (seem), 不太(not too)*, *什么 (any)*, etc.	44.4%
	Evidentials	*据说 (it is said), 应该 (probably), 照理说 (logically)*, *我们医院 (our hospital)*, *我听 X 医生说 (Doctor X told me that), 一般情况下 (normally),* etc.	16.7%
	Tag questions	*是不是 (whether), 好吗 (all right), 是吧 (does it/can I), 对吗 (right), 好不好 (will you)*, *对不对 (is not it),* etc.	25.0%
	Subjectivizers	*要我说 (if you want my opinion), 我个人认为 (personally), 在我看来 (in my opinion), 我觉得 (I think)*, etc.	13.9%
Illocutionary mitigation	Politeness markers	*请 (please)*, *咱 (we/our)*, *咱们 (we/us)*, *请问 (excuse me), 谢谢 (thank you)*, *不客气 (you are welcome)*, *抱歉 (sorry),* etc.	17.1%
	Terms of address	*叔 (uncle), 大娘 (aunt)*, *您 (you)*, *医生 (doctor),* etc.	50.5%
	Disclaimers	*没什么别的意思 (I mean nothing else), 这也不是说 (this is not to say that), 我也不太懂 (I do not understand),* etc.	9.5%
	Supportive utterances	*能问一下 (can I ask), 打扰一下 (excuse me), 有个问题 (have a question), 因为 (because)*, etc.	22.9%
Perlocutionary mitigation	Simple anticipation	*可能你不太理解… (you may not understand that…)*, *说出来你可能不信… (you are not gonna believe that…),* etc.	24.1%
	Concern showing	*您别担心 (please do not worry), 是不是挺疼的 (is that very painful)*, *您放轻松 (please take it easy)*, etc.	48.3%
	Penalty taking	*再动就更严重了 (it will be worse if you move around again), 如果你不重视, 有可能会….(If you do not pay attention to them, they may cause…),* etc.	10.4%
	Direct dissuasion	*建议 (suggest), 避免 (avoid), 尽量少 (as little as possible), 注意 (pay more attention to)* etc.	17.2%

### Positive face management from propositional mitigation

On the basis of Goffman’s definition for face, Spencer-Oatey argued that face was closely related to values of individuality and society, which incorporated the quality face and the social identity face. The former referred to the positive evaluation of the communicators’ personal qualities, such as capacity, aptitude and appearance. The latter indicated that a person’s social identity and social role could be actively maintained and recognized by others. Quality face was strongly associated with self-esteem, and social identity face was closely interrelated with social value orientation (Spencer-Oatey, 2002). In therapist-client conversations, the cognitive-pragmatic function of propositional mitigation was mainly to moderate the force of speech acts with the purpose of saving the quality face and/or the social identity face. Propositional mitigation in therapeutic sessions played a vital role in positive face management and therapist-client relationship rapport management.

**Table:** 

Extract 1	
1 CL:	dan wo ye mei you ***shenme*** qita zhengzhuang a, but I also not have any other symptoms ugh, ye mei you sangzi fayan ***shenme***de. Huozhe kesou, fashao a. also not have throat sore and so on. or cough, fever ugh. However, I do not have ***any*** other symptoms, and I do not have ***any*** sore throat or cough or fever.
2 TH:	nin ting wo shuo, zhege dongxi a, ta bing***buyiding*** shuo ni jiu you listen I say, this thing ugh, it does not necessarily say you should deyou hen mingxiande yanzheng, birushuo, ni qianjitian ganmaole, have very obvious inflammation, for example you a few day before influenza sangzi teng huozhe ***qianjitian*** you yayinyan, yazhouyan huozhe shi shuo. throat sore, or a few days before have gingivitis, periodontitis or is say. bidouyan, suoyou ni shentide yanzheng, ta dou ***you keneng*** zaocheng sinusitis, all your body’s infoammation, it all have chance cause linbajieyan. wo zheyang shuo nin mingbaile ma? lymphadenitis. I like this say you understand what? You listen to me. About your illness, it ***does not necessarily*** mean that you have to have very Obvious inflammation. For example, ***a few days ago***, you had a cold and sore throat, or ***a few days ago***, you had gingivitis, periodontitis or sinusitis. All of your body inflammation ***may*** cause lymphadenitis. Do you understand what I said?
3 CL:	wo jiushi juede ziji ***you dianer*** zhaoji shanghuo, danshi ye I just fell myself have a little worry heat syndrome, but also mei xiangdao hui zheyang. not think of so like this. I just felt ***a little*** anxious to the point of shang huo (a heat syndrome in traditional Chinese. medicine), but I did not expect it to be like this.
4 TH:	zhege dongxi bingbushi shuo nin hui you hen mingquede yanzheng. this thing not mean say you may have very obvious inflammation. nin ba wo gei ni kaide nayao dou chiwanle, bu tengle weizhi. you need I give you prescribe medicine all eat up, not hurt until. bu tengle zan jiu bu li tale. Danshi na bozi shangde daokou, not hurt we will not brother it but that neck on cut. ***youkeneng*** bian xiao, ye ***youkeneng*** meiyou, danshi wanquan xiaoshide may become small, also may none, but totally disappear ***kenengxing*** hen xiao. chance very little. This does not mean you are going to have definite inflammation. You need to take all the medicine I give you until it does not hurt. If it does not hurt, then we will not bother about it. However, the cut on the neck ***may*** become small or ***may*** stay the same, but there is little ***chance*** for the cut to completely disappear.

Understaters, one of the most regularly employed subtypes of propositional mitigation in therapist-client conversations, moderated potentially unpleasant effects of therapist-client interactive conversations by attenuating the propositional commitment of speakers and enhancing the acceptability of the proposition (Sbisà, 2001). Thaler (2012) argued that understaters mostly did not have specific meanings related to semantics, and it was just a form of language expression used by speakers to modify the state or act by making speakers’ proposition less accurate and precise with the purpose of weakening illocutionary force and unwelcome effect of speech acts. Understaters, applied by both therapists and clients, accounted for a large proportion in the therapeutic conversations in this study.

In Extract 1, the client, a 36-year-old woman who is suffering from thyroid crisis, in thyroid department, raised questions about the therapist’s diagnosis based on her knowledge and life experience, but obviously, the relationship between the client and the therapist was rapport-oriented instead of upsetting and discordant, which was mainly because both of them felicitously applied understaters to show respect for each other’s need for face. The client weakened her doubt by using the understater “*shen me*” (any). Although it did not carry any concrete meaning, it represented that there were certain doubts. By using “*shen me*,” the client not only expressed her views reasonably but also maintained the face associated with the therapist’s social identity face. Put another way, the client seemed to describe her conditions to the therapist literally, but actually understaters implied her doubt on the therapist’s diagnosis. Moreover, the client used “*you dian er*” (a little) to modify her own opinion, which could weaken the authority of her own speech acts and relieve the awkward atmosphere caused by her implied doubt. When faced with the doubt raised by the client, instead of directly using opaque medical science principles, he employed understaters, such as “*bu yi ding*” (does not necessarily), “*you ke neng*” (may) and “*qian ji tian*” (a few days ago), to modify his explanation and made it more acceptable and reasonable for the client to accept his diagnosis while maintaining her quality face. This mitigation language also reduced the possibility of negative effects caused by recondite medical knowledge. Afterward, when reporting the condition to his client, the therapist used understaters, such as “*you ke neng*” and “*ke neng xing*” (chance), to weaken the absolute nature of his description of the client’s illness, which could not only relieve the client’s anxiety but also mitigate his own responsibility and maintain his own need to preserve face.

**Table:** 

Extract 2	
1 TH:	shangwu shi duoshao hao nin hai jizhe ma? morning is which number you also remember what? Do you remember the number from the morning?
2 CL:	eng, shangwu 38, XXX(huanzhe mingzi). uh, morning 38,XXX (client name). wo zhenghao guolai nin xiabanle. I just come you come off work. Uh-huh, No. 38 in the morning, XXX (patient name). When I came over, you were going off your shift.
3 TH:	eng? mei you nide mingzi? ni zai xiang xiang, Hm? not have your name? You again think think, ***yinggai*** ji cuo haole, shi bu shi? may remember wrong number, yes or no? Hm? Not your name? You think it over, it is ***probably*** a mistake, is not it?
4 CL:	o o, wo ***yinggai*** ji cuole, buhaoyisi, shi 48 hao. oh oh, I may remember wrong. Embarrassed, is 48 number. Oh, I ***probably*** misremembered, sorry, it is no. 48.
5 TH:	xing, wo kankana. ***Yinggai*** shi linba zhe kuaide wenti. ok, I see. May is lymph this area problem. OK, let me have a look. It is ***probably*** a lymphatic problem.

According to rapport management theory, evidentials were mainly related to social rights and quality face which was closely connected with self-esteem (Spencer-Oatey, 2002). The usage of evidentials was beneficial to moderate the relationship between therapists and clients by generating interactive meanings, which was conducive to both parties to achieve common communicative goals and thus to build a harmonious therapist-client relationship.

Extract 2 also contains such an example, in this extract, the client was a woman aged 36-year-old who visits thyroid department for heat syndrome. In this therapist-client conversation, “*ying gai*” (probably) was applied three times in total. The first time, “*ying gai*” was used by the therapist for positive maintenance of the client’s need for face with the aim of easing the client’s sense of oppression and offense brought on by his speech. At that time, the therapist was sure that this client had misremembered her number, but he applied evidentials “*ying gai*” instead of speech such as “*keng ding*” (definitely) to send an interactive message that he was not directly blaming the client. In this way, the therapist artfully avoided making his client awkward and ashamed and successfully maintained positive face management. Afterward, the second “*ying gai*” was employed by the client to express her uncertainty for the “correct number” mentioned above. It also embodied her implicit apology for her uncertainty. The last “*ying gai*,” applied by the therapist, made the diagnosis “less defined” literally to ease the client’s emotional tension and shorten the therapist-client psychological distance. In this instance, evidentials were used to motivate the client to recall her correct number, which transformed the client as a hearer into an active conversational participator and maintained the client’s quality of face. Thereafter, the client also applied evidentials as a response to the need for positive face to confirm the therapist’s inference. In the course of meeting the demands for face for both therapists and clients, we found that evidentials in therapeutic sessions embodied significance for negotiating rapport in interpersonal meaning and the establishment of supportive therapist-client relationships. Negotiation or meaning negotiation is a vital issue in the field of sociolinguistics, which generally requires communicators’ endeavors to smooth conversations over and achieve successful interactive goals (Varonis and Gass, 1985). In this extract, we found that evidentials was also a part of nonsemantic negotiation of meaning in therapeutic interactions because three “*ying gai*” (probably) played a very important role in constructing interpersonal relationships, without which the greater risk of conflicts happening in therapist-client conversations would increase sharply and inevitably.

### Mutual social right maintenance from illocutionary mitigation

In rapport management theory, social rights mainly indicate that members of a social group believe they should have certain individual rights and/or social rights, which causes them to have mutual expectations on interaction with each other (Spencer-Oatey, 2002). In the course of interaction, social rights fall into equity rights, the right to seek attention from others, and association rights, the rights of proper involvement in communication. In therapeutic sessions, illocutionary mitigation operates on the speaker’s illocutionary nonendorsement or weak endorsement (Caffi, 1999, 2007; Li, 2008). Illocutionary mitigators are important communicative and pragmatic strategies to make speech acts more effective by mitigating the imposition, such as requesting and refusing, which aims at promoting the possibility and acceptability of a certain illocutionary force. Therefore, the major purpose of illocutionary mitigation was to meet the hearers’ social anticipation and maintain their social rights, particularly equity rights and association rights.

**Table:** 

Extract 3	
1 CL:	***yisheng***, xiang wenyixia, jiushi zhuyuan deshihou daizhe jiuxing, shiba? doctor, want ask, is in hospital time take ok, right? ***Doctor***, I want to ask that when I am in hospital, I need to take it with me, right?
2 TH:	dui. right. That’s right.
3 CL:	haode haode, xiexie yisheng. na wo zhijie qu jiuxingleba? ok ok, thanks doctor. Then I directly go ok? Ok, ok. Thank you, ***Doctor***. Then, will I go there directly?
4 TH:	nage, nage de paidui, ni de xian qu paidui. that, that need line up, you need before go line up. gai zuo jiancha hai de zuo jiancha. ni yao deng nage, should do examine also need do examine. You need wait that, na de deng yihuier. then need wait while. Well, there’s a line. You have to line up first. When you need to have a check-up, you get a check-up. You have to wait for that. That is going to take a while.
5 CL:	na dagai yao deng duo chang shijian? then about need wait how long time? How long does that probably take?
6 TH:	zhongwu, de kan qingkuang, zhege mei banfa yuji. noon, need see situation, this no method expect. ***shu,*** zheyang, ***nin*** zhongwu xiuxi huier, chi ge fan, uncle, so, you noon rest while, have a meal, zai guolai kan kan, bie tai lei … then come see see, not too tired… It depends on the situation, and that cannot be expected at noon. Well, ***Uncle***, ***you*** ought to have a meal and take a lunch break then come back. Do not overexert yourself…

In interpersonal communication, terms of address, one subtype of illocutionary mitigation, were applied commonly and widely, especially in the social context including unbalanced power, status, identity and so on. In the performing of speech acts, the speaker could employ certain terms of address, which seemed to be polite or respectful to the hearer, but, in fact, as a kind of mitigating devices with the aim to implement a certain behavior or convey an intention more effectively (Li, 2012).

In this therapeutic session, the client, 45-year-old, asks the therapist the time to have a check-up in doctor’s office. Both the therapists and the clients applied terms of address to mitigate the imposition of speech acts (Czerwionka, 2012). In this instance, three reasons for the usage of “*yi sheng*” (doctor) as terms of address by the client could be explored by decoding the interactive discourse from the perspective of interpersonal relationship management. First, “*yi sheng*” was employed to show the client’s social status in relation to the therapist and to express his respect for the therapist’s professional expertise. Second, two professional titles were utilized to express the client’s obligation to the therapist for helping him with the current problems. The client unconsciously exercised his interactional association rights to actively build relations with the therapist through the meta-pragmatic function of mitigation. Finally, the application of “*yi sheng*” was a good communication strategy to ease the awkwardness resulting from a steady stream of questions and to mitigate the speech acts of asking and requesting. Terms of address used in this instance successfully helped the therapist meet the client’s social anticipation and assisted the client in attaining social rights and managing obligations to reach a relationship of rapport between the therapist and the client. Additionally, when the client raised questions for the third time, the therapist chose terms of address, such as “*nin*” (you) and “*shu*” (uncle), to express his respect and empathic concern for the client, which bridged the therapist-client psychological distance and maintained the interpersonal rapport. Therefore, terms of address were grasped and applied adeptly, especially in situations where communicators risked interpersonal conflicts, such as in therapeutic sessions, to realize better interpersonal relationship management (Friederike, 1988).

**Table:** 

Extract 4	
1 CL:	dan xianzai hai ting tengde. but now still very painful. But it still hurts now.
2 TH:	yaobu zheyangba, wo geren jianyi nin, yaoshi shizai tengde shoubuliao, or like this, I myself advice you, if indeed hurt unbearable, ***zan*** jiu xian guan guanchang, rang duzi lide dongxi xian. we at once first use enema, let stomach in stuff first. pai chuqu, nin kan zenmeyang? excrete out, you see how about? How about this? I personally suggest that if the pain is unbearable, first, ***we*** try to use an enema and let the stuff in the stomach be excreted. What is your opinion?
3 CL:	pai? excrete? excreted out?
4 TH:	dui, zhe jiandan, zhe jiugen yong nage kaisailu shide. yes, this simple, this just like use that glycerine enema like. Yes, it is simple. It is just like using a glycerine enema.
5 CL:	zhenshi ***xiexie*** nin, zhe nianji dale zhenshi maobing duole, Indeed thanks you, this age greater indeed illness more, lao shang huo. often have fire syndrome. ***Thank you*** very much. With getting older, I begin to have lots of illness easily, especially for “fire syndrome.”
6 TH:	nin na, bie tai danxin, queshi, suizhe nianling bianda, ***zan*** zhege you ah, not too worry, really, with age older, we this changwei rudong nengli hui jianruo, yihou duojia duanlian. gastrointestinal peristalsis ability may weaken, later more exercise. jiu mei wentide. will not problem. Do not worry too much. Indeed, as we get older, ***our*** gastrointestinal peristalsis ability weakens, but more exercise will be beneficial to your health.

Politeness markers as one of vital mitigating devices shared little contribution to delivering the semantic information but highlighted its explicit kindness orientation on interpersonal relationship and made verbal communication and pragmatic force more effective to the hearers. In therapist-client interactive conversations, politeness markers, such as “*qing*” (please), “*xie xie*” (thank you), bore the characteristics of interpersonal connotations and social relations rather than a common courtesy (Terkourafi, 2011).

In extract 4, a 52-year-old woman is waiting for her colonic carcinoma operation in inpatient ward. Her stomach was very painful before the operation, but the operation was scheduled for several days out. The therapist employed the first politeness marker “*zan*” (we) instead of directives to send the client to use an enema. Later, when illustrated why older people were apt to have “fire syndrome,” the therapist applied another “*zan*” (our). In many therapeutic sessions, “*zan*” (we/our) or “*zan men*” (we/us), as politeness markers and empathetic deixis, could improve the mutual-receptivity and affinity of the tenor of conversation by perspective taking. Put another way, the therapist took advantage of a pseudoinclusive strategy to achieve identity empathy to avoid looking so superior in the course of the therapeutic session (Li, 2012). The usage of politeness markers was in line with the social anticipation of the client, which made the client feel the therapist was amiable instead of arrogant (Stephan et al., 2010; Mishra et al., 2022). By this means, psychological distance between the therapist and the client was bridged, and mutual social right was maintained.

### Interactive goal concentration from perlocutionary mitigation

In the course of communication, communicators usually set a clear goal for the anticipated outcome of their interaction. In therapeutic sessions, the leading goal for interactive communication is to cure the illness as soon as possible. Therefore, no matter who, either the therapists or the clients, diverged from the interactive goal, the other participant has the social obligation to remind the other explicitly to concentrate on the interactive goal. Thus, perlocutionary mitigation, concentrating on the hearer’s response caused by a certain speech act, enjoys much more mitigating effects than others, as it explicitly works on the unwelcoming effects of speech acts with clear interactional goals and specific interpersonal management targets.

**Table:** 

Extract 5	
1 TH:	***nin fangqingsong***, wo gei nin jiancha jiancha. you relax, I give you examine examine. ***Please take it easy***. I will examine you.
2 CL:	xing. ok. All right.
3 TH:	wo gei nin kai dian yao nin shi shi, hai shide ba nage I give you prescibe some medicine you try try, still need take that xirou gei ge diao, zheyang caineng man man haoqilai. polyp give cut away, in this way then slow slow be fine. I will give you some medicine first. You still need to remove that polyp. And then you can get better slowly.
4 CL:	daifu na shibushi ting tengde a doctor that yes or no very hurt huh Doctor, ***is not that very painful***?
5 TH:	daniang, ***nin bie danxin*** a, weichuang wei chuang, madam, you not worry ah, minimally invasive minimally invasive, jiu yi huier jiu haole. just a moment just fine. Madam, ***do not worry***. It’s minimally invasive, minimally invasive. It will be ok after a little bit.

Concern showing meant that the speaker kept a watchful eye on the hearer’s mental state and emotional situation with the help of linguistic patterns when he or she performed a certain speech act. In the therapist-client interactions, therapists were the major users of concern showing to soothe feelings of the clients. In this way, concern showing embodied the therapists’ pragmatic empathy to show their emotional inclusivity for the clients (Thomas, 1995; Bennefon et al., 2009).

In this extract, a 51-year-old colorectal carcinoma client concerns about the pain for removing her polyp. Both the therapist and the client expressed concern, a frequently used perlocutionary mitigation, to ease nervousness and worry about the medical examination. The therapist noted the client’s nervousness and anxiety before a routine colon examination, so he employed “*nin fang qing song*” (please take it easy) and “*nin bie dan xin*” (please do not worry) as mitigating devices to shorten the cognitive distance to relieve the client’s stress in the therapeutic environment. The client also directly expressed her concern and fear by saying “*shi bu shi ting teng de*” (is not that very painful), which reflected the communicative goal of the client – asking for psychological encouragement. Through the use of concern about herself, the client implied her desire for affective association rights to meet her interactive goal. We found that in therapist-client interactions, almost all therapists were willing to express their attention and concerns for the clients’ emotional withdrawal because of the fear and pain of a certain illness, and sometimes clients themselves also showed concern for their own condition by using mitigation strategies of concern showing. We also found that the skillful usage of concern showing in the therapeutic session was an “emotional lubricant” and “compliance booster,” especially for older people and younger people.

**Table:** 

Extract 6	
1 TH:	zhongwu, de kan qingkuang, zhege mei banfa yuji. noon, need see condition, this not method predict. shu, zheyang, nin zhongwu xiuxi huier, chi ge fan, zai guolai. uncle, like this, you noon rest a while, have a meal, again come. kan kan, bie tai lei. ***Nin keneng bu ai ting***, women yisheng ye see see, not too tired. You may not like listen, we doctor also deyou chi fan shijian, nin shuo shibushi? have eat meal time, you say yes or no? It depends on the situation, which cannot be expected at noon. Well, Uncle, you take a break and have lunch first, then come back. Do not make yourself tired. ***You might not like what I have to say next***, that is, we doctors also need mealtime and some rest. Do not you think?
2 CL:	dui dui, nimen zhende shi xinkule, wo xian qu chi ge fan, yes yes, you really is toilsome, I first go have a meal, guohui zai lai. ***Nin bie xian wo fan***, wo xiang wen wen later again come. You not dislike I trouble, I want ask ask dagai ji dian? about what time? Yes, yes. You are truly hard working. I will have a meal first and come back later. ***Do not mind me***, but I would like to know what time is probably the best for me to come back?

In the therapeutic sessions, the leading purpose for the interactive communication was to cure the illness as soon as possible. Thus, no matter who, the therapists or the clients, diverged from this interactive goal, the other participant had the social obligation to serve as a reminder to re-concentrate on the interactive goal explicitly. In most of the time, the role of the reminder was played by the therapists. Nevertheless, this explicit reminder could not be expressed too directly, or it might give great offense to the hearer and worsen therapist-client relationship (Lakoff, 1973). By the usage of simple anticipation, the goal-divergence participant could not only recognize the deviation, but also not be offended by the well-intentioned “warning,” so as to better concentrate on the interactional goals and maintain the rapport relationship between the therapists and the clients.

In extract 6, the client, 45-year-old, wants to go through the admission procedures in the inpatient department, but it is the mealtime. Both the therapist and client applied simple anticipation to mitigate their perlocutionary act. At lunchtime, the client rushed over to apply for admission, but the therapist was about to leave for lunch and told the client to return to lunch. In this instance, the therapist applied simple anticipation to predict the unwelcoming effects of refusal—the client might show dissatisfaction when his interactive goal failed. By using “*nin ke neng bu ai ting*” (you might not like what I have to say next), the therapist gave a hint of his imminent refusal, which was a brilliant communicative strategy to mitigate the unwelcoming effects of his speech act. This reached a rapport in the therapist-client relationship and bridged the divergence in their interactive goals. The client has never forgotten the goal of interaction by continuously asking the specific time when he could come back. With the anticipation that his repeated questions probably bothered the therapist, the client applied simple anticipation that aimed at maintaining the rapport relationship with the therapist to relieve the unwelcome effects and imposition of his speech act and to avoid unnecessary conflicts.

**Figure 2:**
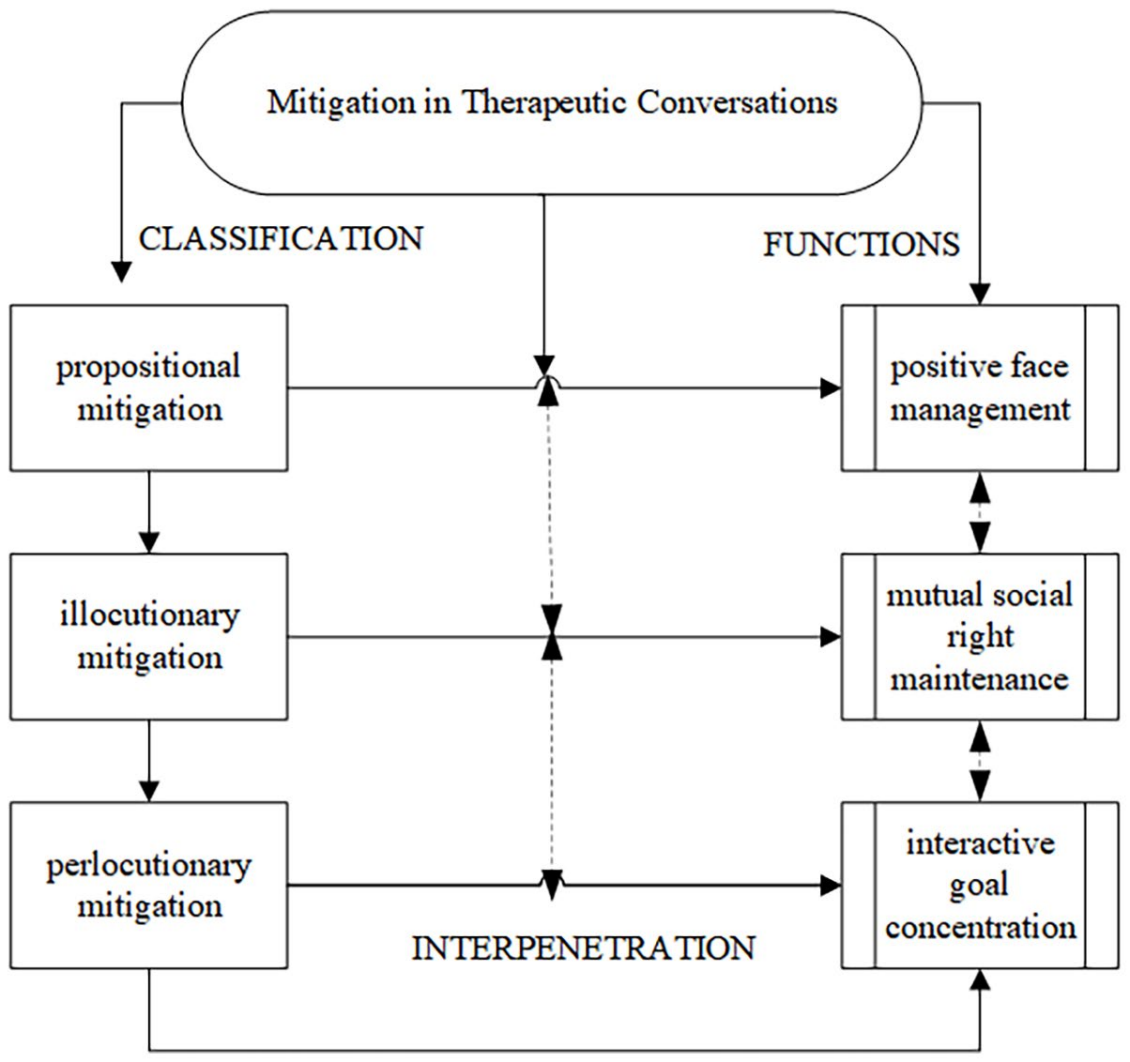
Interpenetration of the cognitive-pragmatic functions of mitigation in therapeutic conversations.

In the interactional practice of mitigation, both therapists and clients were aware of positive face management, mutual social right maintenance and interactive goal concentration to attain rapport in interpersonal relationships. Although, theoretically, we placed disparate emphases on the interpretation of interpersonal management for each type of mitigation, we cannot deny that the interpenetration of the cognitive-pragmatic functions of those mitigating devices served the illocutionary force of a certain speech act (see Figure 2). The first reason was that two or more subtypes of mitigation could occur in one specific therapist-client interactive conversation. For instance, evidentials “*ying gai*” (probably) in propositional mitigation were mainly discussed although terms of address “*nin*” (you) in illocutionay mitigation were also applied felicitously. The cross use of subtypes of mitigation caused interpenetration between their cognitive-pragmatic functions and features. The second was propositional mitigation, illocutionary mitigation and perlocutionary mitigation, to varying degrees, embodied multipragmatic functions and had multiple pragmatic-cognitive approaches. They rarely bore complete segmentation in certain contexts because all of the subtypes of mitigation shared the core characteristic mitigating illocutionary force. The last was that the importance of the three types of management for interpersonal relationships in rapport management theory had overlapping significance for interpersonal relationship management. That is, positive face management could have a great impact on social right maintenance and the concentration on interactive goals and vice versa.

## Discussion

In this article, we have described how therapists and clients manage their therapeutic relationship in a supportive and harmonious way by applying three types and 12 subtypes of mitigating devices in total. We found that mitigation in therapeutic sessions devoted to a rapport relationship *via* managing positive face, maintaining mutual social right and concentrating interactive goal separately or jointly. In therapist-client conversations, disparate mitigating devices were used by either therapists or clients to accomplish the institutional goals and establish a good therapeutic relationship.

Particularly, therapists’ good conversation skills, such as mitigation, were key to successful therapies because they could facilitate honest communication, develop trust, and enhance clients’ willingness to disclose their problematic experiences. We found that in the Chinese context (see Figure 1), tag questions, concern showing and direct dissuasion were mostly used by therapists. Some tag questions were used to show uncertainty but more to negotiate with clients or to induce them to say more for further effective information by creating an opportunity to interact with the clients. As for concern showing, it embodied the therapists’ pragmatic empathy by revealing their emotional inclusivity for their clients. Concern showing explicitly expressed the emotional convergence of the therapists and effectively shortened the therapist-client psychological distance, which directly worked on the clients’ negative affectivity and enhanced clients’ compliance. Finally, in therapist-client interactions, equipped with more professional medical knowledge, therapists applied more direct dissuasion than clients with the purpose of pacification and solidarity building, which was conducive to propose diagnose suggestions and also obtain clients’ compliance. Thus, these three mitigating devices employed most frequently by therapists clearly equipped with benefit to achieve interactive goals and rapport relationship in therapeutic sessions.

Additionally, we also found many clients were adept in using mitigating devices intentionally or unintentionally, which demonstrated understanding of their conditions, established a therapeutic alliance with their therapists, and understood the inner worlds of their therapists and themselves. In Figure 1 we also found that disclaimers, subjectivizers and understaters were employed by clients more frequently during the therapeutic conversations. As for disclaimers, we found that two subtypes of disclaimers, entitlement disclaimers and knowledge disclaimers, were commonly applied by clients when they had discourse incomprehension or absence of corresponding medical knowledge. And subjectivizers were more employed by clients than therapists, and those who availed themselves of subjectivizers would pay more attention to reduce the absolute judgment. While clients applied understaters mainly for the obfuscation of unclear information with the purpose of cutting down their own responsibility.

In general, the current study, together with the applied study of mitigation in therapist-client conversations, demonstrated that means of expression, especially communicative strategy, influenced the practice of therapy. Therapist-clients’ mitigation rather than the ordinary conversations was beneficial to rapport therapeutic relationship by reducing the risk of therapeutic conflicts intentionally and unintentionally. This study may deepen therapists’ understanding of mitigating devices in relation to their importance and effectiveness in therapeutic conversations, thus helping to improve the quality of intervention for their clients. And the insights of clients’ mitigation will supply profound understanding of characteristic of therapist-clients’ conversations, particularly clients’ mitigating devices. Furthermore, the three functions discussed in this study will provide a reference for future studies relating to expressing mitigation during therapeutic interactions in other Chinese contexts.

## Conclusion

This study demonstrated three types and 12 subtypes of mitigators in therapist-client conversations. Propositional mitigation, including understaters, evidentials, tag questions, and subjectivizers, were used to save quality face and social identity face as well as to shorten the interpersonal distance in the course of the therapeutic conversations; illocutionary mitigation, incorporating politeness markers, terms of address, disclaimers and supportive utterances, moderated the pragmatic force and helped the speakers conduct speech acts more effectively and delivered illocutionary force more comprehensively; perlocutionary mitigation, covering simple anticipation, concern showing, penalty taking and direct dissuasion, concentrated on the perlocutionary acts and explicitly worked on the negative effects of speech acts with clear interactional goals. In brief, these three types and 12 subtypes of mitigators in therapist-client conversations served to soften and usually weaken the illocutionary force of a certain speech act with the purpose of lightening unwelcoming effects. From a rapport relationship management perspective, we interpreted the cognitive-pragmatic functions of therapist-client mitigation in the view of positive face management, social right maintenance and explicit concentration on interactive goals. These interpenetrating functions reflected the significance of mitigation as a communicative strategy in the field of establishing a therapeutic rapport relationship from an original and specific point of view. The findings of the research may complement the professional stock of therapist-client interactional knowledge with which therapeutic work runs smoothly.

## Data availability statement

The original contributions presented in the study are included in the article/Supplementary material, further inquiries can be directed to the corresponding author.

## Ethics statement

The studies involving human participants were reviewed and approved by 中国石油大学(华东)外国语学院伦理委员会. The patients/participants provided their written informed consent to participate in this study. Written informed consent was obtained from the individual(s) for the publication of any potentially identifiable images or data included in this article.

## Author contributions

LC designed the study, analyzed the data, and wrote the manuscript. HM and TZ conceived this study, collected the data, and revised the manuscript. LC and HM transcribed the data, reviewed the literature, wrote sections of the manuscript, and revised the manuscript. All authors contributed to the article and approved the submitted version.

## Funding

This research was funded by a grant from the Humanities and Social Sciences Youth Fund of the Ministry of Education of China (Title: *Assessment and intervention of children with autism in Chinese*) (No. 20YJC740008), a grant from the Fundamental Research Funds for the Central Universities (Title: *The cognitive research of autistic children with language disorder*s) (No. 22CX04014B), and a grant from China Postdoctoral Science Foundation, No. 71 General Fund (Title: *A Study on metaphor and metonymy acquisition of Chinese autistic children*) (No. 2022M712151).

## Conflict of interest

The authors declare that the research was conducted in the absence of any commercial or financial relationships that could be construed as a potential conflict of interest.

## Publisher’s note

All claims expressed in this article are solely those of the authors and do not necessarily represent those of their affiliated organizations, or those of the publisher, the editors and the reviewers. Any product that may be evaluated in this article, or claim that may be made by its manufacturer, is not guaranteed or endorsed by the publisher.
